# Nursing Care to Reduce Suicide Risk in Cancer Patients: A Narrative Review of the Literature

**DOI:** 10.3390/nursrep15080265

**Published:** 2025-07-24

**Authors:** Álvaro Borrallo-Riego, María García-Mayo, Irene Gil-Ordóñez, Isabel Domínguez-Sánchez, María Dolores Guerra-Martín

**Affiliations:** 1 Nursing Department, Faculty of Nursing, Physiotherapy and Podiatry, University of Seville, 41009 Seville, Spain; aborrallo@us.es (Á.B.-R.); guema@us.es (M.D.G.-M.); 2 Institute of Biomedicine of Seville (IBiS), 41013 Seville, Spain; 3 Faculty of Nursing, Physiotherapy and Podiatry, University of Seville, 41009 Seville, Spainiregilord@alum.us.es (I.G.-O.)

**Keywords:** nursing, nursing care, suicide, suicide prevention, neoplasms

## Abstract

**Background**: Cancer is one of the leading causes of morbidity and mortality worldwide and in Spain. Individuals with cancer are at a higher risk of suicide compared to the general population due to both general and disease-specific risk factors. **Objective**: To update knowledge on nursing care measures to address the risk of suicide in cancer patients. **Methods**: A narrative review was conducted by searching PubMed, WOS, Scopus, and CINAHL during February and March 2025. The inclusion criteria comprised original qualitative, quantitative, and/or mixed-methods studies related to the topic of the review. **Results**: Of the 289 identified studies, 23 were selected. Twelve studies of cancer patients, ten studies of healthcare professionals, and one study of caregivers and survivors were included. Regarding suicide risk factors, eight studies addressed demographic aspects, fifteen socio-economic factors, twenty-one psycho-emotional factors, and seventeen physical factors. Key risk factors included male sex, advanced age, social isolation, lack of social support, hopelessness, and physical deterioration. Seventeen studies highlighted the need for continuous and comprehensive nursing care using validated tools for systematic assessment of suicide risk. Eight emphasised the importance of ongoing training in suicide prevention, which is essential for developing communication skills and improving therapeutic relationships. Five studies underscored the relevance of a holistic approach that addresses the physical, emotional, social, and spiritual dimensions of patient care. Six extended this approach to include family members and caregivers. **Conclusions**: Suicide risk in cancer patients is associated with multiple risk factors. Emotional support and a comprehensive, continuous nursing approach—based on systematic assessments, specialised training, and a holistic focus—are key to effective suicide prevention.

## 1. Introduction

Currently, cancer is one of the leading causes of morbidity and mortality worldwide. In Spain, it is estimated that in 2025 there will be more than 296,103 new cases, representing a 3.3% increase compared to 2024. Furthermore, the number of new diagnoses is projected to rise to 28 million by 2040. This increase has been associated with multiple factors, including population ageing and exposure to risk factors such as tobacco, alcohol, obesity, and sedentary lifestyles [1,2].

Cancer patients face a higher risk of suicide compared to the general population, as both their physical and mental health are affected [3,4,5]. Indeed, various authors report that suicide rates in this population can be four to five times higher than in individuals without cancer [6,7,8,9,10]. This suicide risk may persist throughout the entire care trajectory [11,12].

It should be noted that suicide rates among cancer patients are not uniform but vary according to cancer type and stage. Globally, the overall incidence of suicide deaths among cancer patients has been reported at 39.72 per 100,000 persons per year [13,14].

Cancer patients encompass both the general risk factors for suicide present in the general population and others specific to their clinical situation. In this regard, risk factors can be observed at the demographic level (such as sex, age, or marital status), at the socio-economic level (such as loss of social support, difficulties in social interactions, or changes in employment status), at the psycho-emotional level (such as depression, hopelessness, or fear and anxiety), as well as at the physical level (such as chronic pain), which may affect the individual’s autonomy. Chronic pain is a key consideration, as it affects daily activities, social relationships, sleep quality, and mood. This exacerbates the risk of suicide not only during the disease but also after cancer remission. Furthermore, emotional disorders can affect not only patients but also their families and caregivers; thus, psychological and emotional care should be part of comprehensive cancer care [14,15,16].

Given the high risk of suicide in oncology patients, it is essential to implement measures to address and prevent this risk, including the promotion of protective factors. Protective factors reported include dimensions of personality such as adequate self-esteem, cognitive flexibility, controlled impulsivity, emotional stability, and appropriate psychological coping resources [17]. These factors promote individual resilience to suicide [18]. Social and family factors also play a protective role, as strong social relationships, cultural integration, and family support enhance a person’s ability to withstand suicidal behaviour [19]. The family significantly influences the development of emotional and social skills, especially during emotional crises. Therefore, in cases of suicide risk, the family becomes a fundamental pillar in managing and preventing risk factors and recognising warning signs such as isolation, loss of appetite, or discontinuation of daily activities [20].

Nurses provide continuous holistic care to cancer patients, making their role crucial in risk assessment and prevention. Nursing care can be guided by various nursing models, such as Callista Roy’s Adaptation Model, which posits that adaptive responses promote the person’s integrity by considering his capacity to adapt to environmental changes and challenges [21]. These adaptive aspects should be considered in oncology patients and reinforced by specialised oncology nurses, who have skills not only in symptom management and pain control, but also in strategies to improve quality of life and facilitate effective communication with patients, families, and healthcare teams, thus promoting biopsychosocial care [22,23,24]. However, several studies report that nurses face challenges in assessing suicide risk, noting that systematic suicide risk screening programmes are underutilised in cancer care [25,26,27].

Considering the above, this study aims to identify factors associated with suicide risk in oncology patients and to analyse nursing care strategies aimed at preventing and reducing this risk.

## 2. Materials and Methods

### 2.1. Study Design and Search Strategy

A narrative review was conducted by searching PubMed, WOS, Scopus, and CINAHL during February and March 2025, following the recommendation of Urrútia and Bonfill [28]. The studies’ search strategy was defined by consensus between the authors to avoid bias for not including relevant studies [29], namely: [(“Nursing care” OR Nurs*) AND (Suicide* OR “Suicide Prevention”) AND (Neoplasm* OR Cancer)].

### 2.2. Eligibility Criteria

The eligibility criteria for the studies were presented with the greatest transparency and clarity to control selection bias [30]. Studies were included if they (1) followed a quantitative, qualitative, or mixed-methods design; (2) involved cancer patients, caregivers of cancer patients, or nurses; and (3) reported outcomes related to suicide risk factors in cancer patients and/or nursing care strategies aimed at preventing or reducing this risk. Protocols or projects in which these outcomes were neither measured nor reported were excluded. No restrictions were applied regarding language or publication date.

### 2.3. Selection of Articles

Studies were selected in four stages. The first stage consisted of searching and locating studies in the databases using the predefined search strategy. The second stage comprised the elimination of duplicates, which was managed using Covidence software (https://www.covidence.org/). In the third stage, Covidence was also used to manage the independent screening of titles, keywords, and abstracts by two authors (M.G.-M. and I.G.-O.), selecting those consistent with the review subject and eligibility criteria. The fourth stage consisted of a critical reading of the full texts of the selected articles. Any disagreements were resolved by consensus with a third author (A.B.-R.).

### 2.4. Data Extraction

Two reviewers (M.G.-M. and I.G.-O.) independently extracted the data from the selected studies using a standardised data extraction form designed following the recommendations of Del Pino et al. [29]. The extracted information included (a) author/s; (b) study design and methods; (c) study period, sample, and country; (d) suicide risk factors; (e) nursing care. All disagreements regarding data extraction were resolved by consensus with a third author (A.B.-R.). The extracted data were subsequently discussed and synthesised collaboratively by all authors.

### 2.5. Data Analysis

A narrative synthesis approach was used to analyse and integrate the findings from the selected studies. Reported suicide risk factors were systematically identified, compared, and grouped into thematic categories based on recurring concepts and conceptual similarities across studies. Similarly, nursing care strategies aimed at mitigating suicide risk were summarised, highlighting common interventions, preventive measures, and approaches described in the literature. Patterns and discrepancies between studies were discussed collaboratively among the authors to refine and validate the thematic synthesis. This narrative approach allowed for the integration of both qualitative and quantitative findings.

## 3. Results

### 3.1. Description of the Studies and Characterictics of the Sample

**Figure 1:**
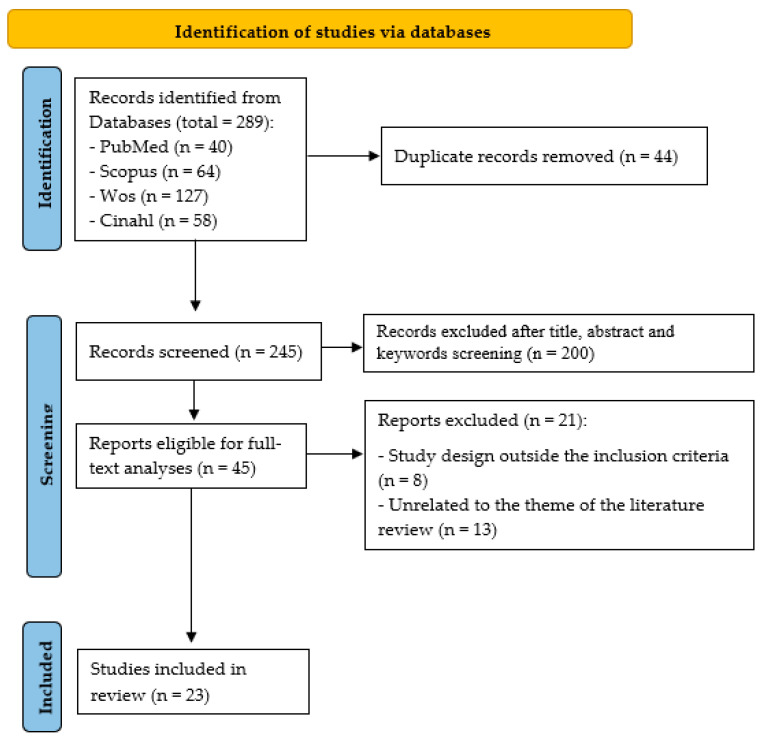
Flow diagram of the study.

Of the 289 studies initially identified, 23 were selected as consistent with the review objective and eligibility criteria (Figure 1).

The specific characteristics of the studies are detailed in Table 1. Regarding study design, twelve studies were quantitative [31,32,33,34,35,36,37,38,39,40,41,42], eight were qualitative [23,43,44,45,46,47,48,49], and three employed a mixed-methods approach [50,51,52]. In terms of data sources, the quantitative studies primarily relied on scales and/or questionnaires in nine cases [31,32,33,36,37,38,39,40,42], while the remaining ones used secondary data derived from official records [34,35,41]. All qualitative studies used interviews as their main data source. For the mixed-methods studies, two combined questionnaires and/or scales for the quantitative component with interviews for the qualitative component [50,52]. In the study by Men et al. [51], forensic reports were used as secondary data for the quantitative analysis, while suicide notes were examined using a qualitative approach.

Regarding the study population, twelve studies included patients with cancer [31,32,33,34,35,37,38,40,41,42,49,51]. Ten focused on the perspectives of healthcare professionals who work with cancer patients [23,36,39,43,44,45,46,47,48,50], while one study explored the views of caregivers and cancer survivors [52].

In terms of sex distribution, three studies included only female participants [40,45,52]. In eleven studies, the proportion of women was higher than that of men [23,32,33,36,37,38,39,42,43,48,50], whereas in the remaining nine studies, male participants were more prevalent [31,34,35,41,44,46,47,49,51].

Concerning the age of the participants, three studies included individuals under the age of forty [23,43,50]. In nine studies, participants ranged in age from forty to sixty years [33,36,37,38,39,40,48,49,52]. Two studies involved populations over the age of sixty [31,35], and in the remaining nine studies, age-related data were not reported [32,34,41,42,44,45,46,47,51].

### 3.2. Study Outcome

#### 3.2.1. Demographic, Socio-Economic, Psycho-Emotional, and Physical Factors Associated with Suicide Risk in Cancer Patients

Eight studies referred to demographic-level risk factors [23,31,34,35,37,41,50,52]. Of these, four identified male sex as a recurrent risk factor [31,34,35,50], while three highlighted white race [34,35,41]. These same studies also reported older age as a contributing factor. However, two studies emphasised particular vulnerability among adolescents and young adults [37,52]. Marital status was described as a risk factor in two studies, specifically noting being single, divorced, or widowed [41,52]. Place of residence also emerged as a relevant factor, with rural living associated with a higher risk [34]. A low level of educational attainment was reported as a risk factor in two studies [23,52].

Fifteen studies addressed socio-economic risk factors [31,32,33,34,35,36,38,41,43,44,45,47,48,49,51]. In eight of these, lack of social support was emphasised, with difficulties in social interactions and deterioration of family relationships linked to an increased risk [32,34,43,44,47,48,49,51]. Social stigma surrounding mental illness and suicide was also identified as a significant contributor, as it may deter individuals from seeking help [33,38]. Nine studies highlighted the lack of access to mental health services and professionals as a key issue, along with the risks associated with economic instability, including poverty, unemployment, and job loss [31,32,34,35,36,41,43,45,51].

All studies except two [34,50] referred to psycho-emotional risk factors. Emotional distress was consistently identified as one of the main risk factors, with the potential to trigger anxiety, depressive moods, irritability or aggression, feelings of hopelessness, loss of meaning or control, and fear stemming from perceived threat [23,31,33,36,42,43,51]. These emotional states may contribute to diminished self-efficacy and ineffective coping, both at the time of diagnosis and during treatment, thereby increasing vulnerability. This vulnerability is further exacerbated in individuals with a prior history of mental illness or suicide attempts, as well as in those with comorbid active psychiatric conditions [23,32,35,37,38,39,40,41,44,45,46,47,48,49,52].

Seventeen studies discussed physical risk factors [23,31,32,33,34,35,36,40,42,44,45,46,47,48,49,50,51]. In this regard, the studies predominantly reported deterioration in health status and symptomatology. Pain was highlighted as one of the principal causes of physical suffering and distress. Fatigue and weakness were also described, with some cases leading to physical decline or incapacity. One study specifically pointed to the lack of physical activity as an additional physical risk factor [32].

#### 3.2.2. Nursing Care to Reduce the Risk of Suicide in Oncology Patients

The studies included in this review highlight a series of concrete nursing strategies aimed at preventing suicide risk in cancer patients:

(1) Systematic suicide risk assessment: Seventeen studies emphasised the importance of applying validated tools to assess suicide risk. Instruments such as the Columbia-Suicide Severity Rating Scale (C-SSRS) and the Plutchik Suicide Risk Scale are recommended for baseline assessment and periodic reassessment—ideally every three months or following major clinical or psycho-social changes [31,33,34,35,36,37,41,42,43,44,45,46,47,48,49,50,52]. These tools should be complemented by clinical interviews and regular review of patient records to ensure early identification of risk [35,48,52].
(2) Professional training and communication skills development: Eight studies underlined the need for continuous professional development focused on suicide risk recognition, empathic communication, and ethical support practices [35,36,39,40,43,44,45,46]. Proposed strategies include annual workshops, case simulations, and structured communication protocols to improve nurses’ capacity to detect warning signs such as hopelessness or existential distress [35,39,43,45].
(3) Therapeutic and supportive nursing interventions: Several studies reported the effectiveness of specific nursing interventions to alleviate emotional and physical suffering. These include progressive muscle relaxation, therapeutic walking, aromatherapy, behavioural activation therapy, and problem-solving approaches. Such techniques are seen to reduce anxiety, depressive symptoms, and physical discomfort while enhancing quality of life [40,43,45].
(4) Integration into routine clinical practice and interdisciplinary coordination: Some authors advocated for embedding suicide risk assessment into standard nursing documentation and shift reports to promote consistency in detection and follow-up [35,41,46]. Coordination with mental health professionals and social workers was also considered essential to ensure timely and effective response to high-risk cases [45,47].
(5) Family and caregiver support: Eight studies extended suicide prevention strategies to include family members and caregivers, especially in cases involving advanced cancer or young survivors. Interventions included emotional support for caregivers, identification of their own suicide risk or psychological distress, and provision of coping tools and referrals to specialised care when necessary [32,38,47,48,49,50,51,52].

**Table 1:** Characteristics of the selected studies.

Authors	Study Design/Methods	Study Period/ Sample/Country	Suicide Risk Factors	Nursing Care
Schwinn et al. [50]	Mixed-methods study. Methods: On the qualitative side, semi-structured interviews were conducted focusing on work experience in oncology, experiences with suicidal behaviour, and suicide risk assessment. The average interview duration was 39.47 ± 8.35 min. Content analysis of the interview transcripts was carried out using MAXQDA R22.7 V5 software. For the quantitative data collection, a questionnaire was administered. Data analysis was performed using R Statistics V.4.3.1.	Period: Not reported. Sample: 20 healthcare professionals from an oncology unit (female: 70%; mean age: 41.45 ± 9.7 years). Country: Germany.	Demographic: Male sex. Socio-economic: Not reported. Psycho-emotional: Not reported. Physical: Worsening of physical symptoms.	The authors highlight the need for adequate training, clear guidelines, validated tools, and referral pathways to support the identification and management of suicidal behaviour. They recommend systematic risk assessment (e.g., C-SSRS) and fostering a trusting relationship with the patient and their family.
Espuig et al. [31]	Quantitative study. Methods: Questionnaires used: SF-36, B-IPQ, HADS and Plutchik Suicide Risk Scale. Descriptive analysis of socio-demographic and clinical variables was conducted using SPSS 28.0. Pearson correlations were used to examine associations, and linear regression analysis was performed to identify predictors of suicide risk.	Period: 2019–2024 Sample: 71 patients (female: 29.94%; mean age: 65.18 ± 12.02 years) Country: Spain	Demographic: Single, widowed, and childless. Socio-economic: Lack of social support, limited access to healthcare, and economic instability. Psycho-emotional: Emotional distress and perceived threat. Physical: Pain.	It is essential to address both physical and emotional needs to reduce anxiety and stress. Suicide risk should be assessed systematically. Establishing a trusting relationship and acting as a link between the patient, family, and professionals is key. Effective communication—clear, supportive, and accessible—helps decision-making, coping, and self-care.
Öztürk & Hiçdurmaz [23]	Qualitative study. Methods: Data were collected both in person and online, and subsequently via email from individuals who had previously participated. Statistical analysis was performed using SPSS and SPSS AMOS v.20.0. Continuous variables were assessed using the median and interquartile range, while categorical variables were presented as frequencies and percentages.	Period: 2021–2022 Sample: 252 oncology nurses (female: 91%; mean age: 29 years) Country: Türkiye	Demographic: Low educational level. Socio-economic: Poverty and unemployment. Psycho-emotional: Depression, anxiety, emotional distress, and presence of psychiatric illness. Physical: Associated physical discomfort.	It is important to identify suitable psycho-social resources for each individual. This requires nursing staff to be trained in empathic communication. The Perceived Effectiveness in Suicide Risk Management Scale is recommended to guide training and evaluate protocols.
Zhang et al. [32]	Quantitative study. Methods: Use of the SRI-25 questionnaire. Descriptive techniques, univariate tests, and Pearson correlations were applied for analysis. Additionally, a serial multiple mediation model (PROCESS Model 6) was used to assess mediation effects.	Period: January to March 2022 Sample: 287 cancer patients (female: 58.2%; mean age: not reported) Country: China	Demographic: Not reported. Socio-economic: Lack of social support; unemployment. Psycho-emotional: Lack of coping tools. Physical: Lack of physical activity.	The authors recommend providing continuous emotional support, reinforcing self-care and symptom management strategies, and addressing feelings of burden or guilt. Involving family caregivers is also key to strengthening the patient’s support network and sense of purpose.
Zheng et al. [33]	Quantitative study. Methods: The following scales were used: SSQ, MINI Suicide Subscale, PHQ-9, GAD-7 and DT Statistical data analysis was conducted using SPSS v.27.0, PASS v.2021, and Microsoft Excel v.2016.	Period: March 2018–August 2023 Sample: 9703 cancer patients (female: 73.47%; mean age: 49.69 ± 11.59 years) Country: China	Demographic: Not reported. Socio-economic: Changes in social roles. Psycho-emotional: Depression and anxiety. Physical: Pain, fatigue, and weakness.	The authors highlight the Single Suicide Question (SSQ) as a useful initial tool for detecting suicide risk in cancer patients. However, they stress the need to develop simple, concise oncology-specific methods to improve response quality. Nurses should be trained in using these assessment tools.
Hu et al. [34]	Quantitative study. Methods: Data were obtained from the North American Cancer Incidence Survival Registry (NA-CISR) and the National Centre for Health Statistics. Two analyses were conducted: 1. Standardised mortality ratios; 2. Risk ratio estimation using regression analysis.	Period: 2000–2016 Sample: 16,954,604 cancer patients (female: 48.5%; mean age: not reported) Country: United States	Demographic: Male sex, non-Hispanic white ethnicity, advanced age, rural residence. Socio-economic: Lack of healthcare coverage under Medicare. Psycho-emotional: Not reported. Physical: High symptom burden.	The authors highlight the importance of suicide risk screening and personalised psycho-oncological interventions, alongside developing clear clinical guidelines. These measures promote early intervention, improve access to support, and strengthen resilience.
Katayama et al. [35]	Quantitative study. Methods: Data were collected based on information identified in Medicare. Clinical, demographic, and mental health variables were gathered, along with social vulnerability index and access to mental health services. Analysis included descriptive statistics, chi-square tests, multivariable logistic regression, and variable interaction analysis.	Period: 2004–2016 Sample: 382,266 cancer patients (female: 47.6%; mean age: 72.0 years) Country: United States	Demographic: Male, single, white ethnicity. Socio-economic: Social isolation, limited access to mental health care. Psycho-emotional: Depression, anxiety, bipolar disorder. Physical: Treatment-related physical issues and disability.	Systematic detection and screening using self-report questionnaires such as PHQ-9, BHS, and the Hospital Anxiety and Depression Scale, complemented by regular review of medical records to identify risk factors. Enhancing training and education on suicide risk factors in cancer patients is essential, along with ensuring timely referrals to mental health professionals.
Öztürk & Hiçdurmaz [43]	Qualitative study. Methods: Data were collected through individual, face-to-face semi-structured interviews. A content analysis was conducted. The COREQ guidelines were followed for report preparation. The average duration of the interviews was 39.3 ± 7.1 min.	Period: July–October 2019. Sample: 33 oncology nurses (female: 90%; mean age: 36 ± 8.7 years). Country: Türkiye.	Demographic: Not reported. Socio-economic: Job loss, difficulties in social interaction, and deterioration of family relationships. Psycho-emotional: Depressive mood, aggression, feelings of despair. Physical: Not reported.	Develop training to improve suicide risk recognition and management. Nurses should systematically assess risk, promote effective communication, and support patients’ hope and spirituality. An institutional culture fostering prevention and timely psycho-social referrals is essential.
Men et al. [51]	Mixed study. Methods: Quantitative data were collected from reports prepared by the Hong Kong Coroner’s Court. Qualitative data collection involved a qualitative analysis of suicide notes using a grounded theory approach to identify and code motivations for suicide.	Period: 2012–2017 Sample: 551 individuals with a history of cancer (female: 34.12%; mean age: Not reported) Country: China	Demographic: Not reported. Socio-economic: Concerns related to work and social relationships. Psycho-emotional: Depression, anxiety, feelings of guilt and remorse. Physical: Associated physical discomfort.	The authors describe that suicide prevention should not only focus on the cancer patient but also on their family members and/or caregivers, providing follow-up services that offer emotional and social support. It is key that nurses address the mental, social, and physical needs, thereby delivering a holistic and comprehensive approach.
Senf, Maiwurm & Fettel [36]	Quantitative study. Methods: Quantitative data were collected using an online questionnaire consisting of 48 closed and open-ended questions, distributed via email. Quantitative data analysis was performed using IBM SPSS v.23.0. Qualitative data were evaluated through content analysis following Mayring’s method, using the software QCAmap.	Period: 2017–2018. Sample: 354 healthcare professionals working with cancer patients (female: 77.1%; mean age: 47.8 ± 11.5 years). Country: Germany.	Demographic: Not reported. Socio-economic: Social isolation. Psycho-emotional: Feelings of loss of control and autonomy, anxiety, depression. Physical: Pain, physical disability.	The authors highlight the importance of nurse training in communication and suicide risk management to effectively identify and support oncology patients at risk. Nurses should develop specialised knowledge and assessment skills and be able to refer patients to specialists when necessary.
Walker et al. [37]	Quantitative study. Methods: Data were collected through the Scottish National Health System Cancer Registry. The Hospital Anxiety and Depression Scale (HADS) was also administered. All data analyses were performed using Stata 16.	Period: 2008–2012. Sample: 2217 cancer patients with comorbid major depression (female: 80%; mean age: 60 ± 8 years). Country: United Kingdom.	Demographic: Young people. Socio-economic: Not reported. Psycho-emotional: Depression. Physical: Not reported.	The authors describe the need to implement depression screening programmes for the assessment and management of suicide risk in oncology patients.
Yang et al. [38]	Quantitative study. Methods: Data were collected using the following scales: IRI, CBI, SDS, SIS, and BSI. All data analyses were conducted using EpiData Entry version 3.1 and SPSS version 25.0.	Period: 2019–2021. Sample: 358 cancer patients (female: 52.51%; mean age: 55.2 ± 3.8 years). Country: China.	Demographic: Not reported. Socio-economic: Social stigma. Psycho-emotional: Insufficient self-efficacy, depressive symptoms. Physical: Not reported.	The authors emphasise the importance of personalised care that helps patients and families recognise their strengths, alongside psycho-social counselling, health education, and palliative care, when necessary, to improve quality of life and reduce suicide risk.
Granek et al. [44]	Qualitative study. Methods: In-depth interviews were conducted. Interview transcripts were analysed using line-by-line systematic coding following Grounded Theory principles to identify emerging themes. NVivo 10 software was used to organise, classify, and code the data in a structured manner.	Period: Not reported. Sample: 61 healthcare professionals (female: 32.79%; mean age: not described). Country: Israel.	Demographic: Not reported. Socio-economic: Lack of social support. Psycho-emotional: Emotional distress, hopelessness, history of prior mental disorders. Physical: Physical deterioration related to disease progression.	The authors highlight the following care measures: 1. Promoting communication with the patient to ensure greater sensitivity in the choice of words when interacting with the patient and their family, emphasising essential skills such as empathy and humanisation. 2. Monitoring warning signs, including body language and expressions or desires of death from patients.
Senf, Maiwurm & Fettel [39]	Quantitative Study Methods: Data were collected using a 48-item online questionnaire comprising both closed and open-ended questions, distributed via email. Quantitative data were analysed using SPSS version 23.0. Qualitative responses were assessed through content analysis following Mayring’s method, employing the QCAmap software.	Period: December 2017–February 2018. Sample: 354 healthcare professionals working with cancer patients (women: 77.1%; mean age: 47.8 ± 11.5 years). Country: Germany.	Demographic: Not reported. Socio-economic: Not reported. Psycho-emotional: Pre-existing psychiatric illness (depression), high levels of distress. Physical: Not reported.	The authors propose the following measures to improve care: (1) implement psycho-oncological training alongside additional education focused on managing suicidal tendencies; (2) introduce the KoMPASS programme, which centres on communication training to promote positive changes in nurses’ communicative behaviours and attitudes, thereby enhancing professional–patient communication.
Granek et al. [45]	Qualitative Study Methods: Grounded theory was employed to analyse interviews conducted with oncology nurses from two academic oncology centres in Israel. Categories were defined concerning the barriers encountered, therapeutic approaches, and perceptions regarding the management of psychological distress and suicidal behaviour. Software: NVivo 10.	Period: Not reported. Sample: 20 nurses (women: 100%; mean age: not reported). Country: Israel.	Demographic: Not reported. Socio-economic: Lack of availability and accessibility of mental health resources. Psycho-emotional: Stigma surrounding mental health care. Physical: Physical pain.	1. Establish effective communication with key professionals (psychologists, social workers) to assess and manage suicide risk individually, providing early emotional support. 2. Promote continuous training on identifying and managing suicide risk, focusing on pain, distress, and suicidal thoughts. 3. Implement regular suicide risk assessments and tailored intervention protocols in oncology care.
Granek et al. [46]	Qualitative Study. Methods: Interviews were conducted using a grounded theory design. The analysis involved identifying emerging categories and patterns from the collected data. Software: NVivo 10.	Period: November 2015 to June 2016. Sample: 61 oncology health professionals (women: 32.79%; mean age: not reported). Country: Israel.	Demographics: Not reported. Socio-economic: Not reported. Psycho-emotional: Expression of distress, hopelessness, mental suffering. Physical: Physical suffering related to illness.	Training healthcare professionals in suicide risk detection and prevention using tools like PHQ-9 or BHS is essential. An ethical approach respecting patient autonomy and providing emotional support should be promoted. Creating spaces for professional reflection and ensuring early referral of at-risk patients to mental health specialists are also crucial.
Granek et al. [47]	Qualitative study. Methods: Individual, semi-structured, in-depth interviews were conducted using a grounded theory design. The sample was purposive and focused. NVivo 10 software was used for data management and analysis.	Period: Not reported. Sample: 61 oncology healthcare professionals (women: 32.79%; mean age: Not specified). Country: Israel.	Demographic: Not reported. Socio-economic: Lack of social support. Psycho-emotional: History of mental health disorders; hopelessness and perceived lack of meaning in life; difficulties coping with diagnosis and treatment. Physical: Chronic pain and functional decline.	Suicide risk should be assessed through direct questions about suicidal ideation and by recognising verbal and non-verbal signs. Creating a safe, empathetic environment that encourages emotional expression is essential. Additionally, documenting psychiatric history and offering coping strategies is important. Involving family members strengthens prevention and care.
Granek et al. [48]	Qualitative study. Methods: Interviews were conducted based on grounded theory. Categories were developed related to the understanding, assessment, and management of suicidal ideation, as well as factors influencing clinical decision-making and ethical judgment. NVivo 10 software was used.	Period: Not reported. Sample: 61 professionals [oncologists: 23; social workers: 18; nurses: 20] (women: 85.2%; mean age: 47.7 years). Country: Israel.	Demographic: Not reported. Socio-economic: Lack of social support. Psycho-emotional: Fear of being a burden, history of mental illness, delivering bad news. Physical: Physical pain and suffering, disease relapse or progression.	Nurses should identify signs of distress and suffering, as well as suicide risk. This involves conducting individualised assessments and enhancing training to promote coping techniques. They should also encourage open communication about death and patient wishes to identify potential risks, including involving the family in these discussions. Early intervention is recommended for at-risk patients.
Sun et al. [40]	Quantitative Study. Methods: Data were collected using the following scales: CES-D, BSI, and the World Health Organization Quality of Life Questionnaire. Data analysis was conducted using IBM SPSS Statistics. Independent *t*-tests were used to compare means of continuous variables, and the chi-square test was applied to compare categorical variables between two groups.	Period: September 2014–September 2015. Sample: 87 breast cancer patients undergoing chemotherapy (women: 100%; mean age: 54.1 ± 7.7 years). Country: China.	Demographics: Not reported. Socio-economic: Not reported. Psycho-emotional: Depression, hopelessness, mental exhaustion, pre-existing mental illness, and history of suicide attempts. Physical: Physical pain, physical deterioration due to disease progression.	Teach cancer patients muscle relaxation techniques and promote therapeutic walking to help reduce depression, improve quality of life, and alleviate physical pain and negative emotions. Additionally, other methods such as aromatherapy, behavioural activation therapy, and problem-solving therapy can be used. Training in beneficial exercise programs for these patients is also necessary.
Zhang et al. [49]	Qualitative Study. Methods: Individual face-to-face interviews were conducted. The data translation followed a back-translation procedure, and thematic analysis was performed according to Braun and Clarke.	Period: April to November 2014 Sample: 32 patients with stomach cancer (women: 15.6%; mean age: 56.8 years). Country: China.	Demographic: Not reported. Socio-economic: Caregiver fatigue and lack of social support. Psycho-emotional: Lack of coping skills and hopelessness. Physical: Physical problems due to poor treatment adherence.	Evaluate modifiable risk and protective factors in each patient by: (a) implementing protocols for suicide risk assessment and management, including appropriate training; (b) facilitating communication between patients and families to strengthen family support as a protective factor; and (c) coordinating with other professionals to provide comprehensive care.
Klaassen et al. [41]	Quantitative Study. Methods: Data were collected from the Surveillance, Epidemiology, and End Results (SEER) database and the National Center for Injury Prevention and Control. Statistical analyses were conducted using SAS version 9.4.	Period: 1988–2010. Sample: 1,178,000 bladder cancer patients (women: 286,001, 24.27%; mean age: not reported). Country: United States.	Demographic: Male sex, white race, age over 80, and single/divorced/widowed marital status. Socio-economic: Low socio-economic status. Psycho-emotional: History of psychiatric illness or abuse. Physical: Not reported.	The authors emphasise the importance of clinical professionals being aware of suicide risk factors in patients diagnosed with cancer and employing mental health intervention strategies to manage them effectively and improve quality of life. Nursing staff should conduct routine distress screenings for all patients to identify early signs and enhance psychological care.
Park, Chung & Lee [42]	Quantitative Study. Methods: Data were collected using structured questionnaires: Pain in advanced cancer patients, HADS, ECOG and Suicide Risk Assessment Subscale of the Mini International Neuropsychiatric Interview (MINI). Data analysis was performed using SPSS version 18.0.	Period: Not reported. Sample: 457 advanced cancer patients (women: 74.5%; mean age: not described). Country: South Korea.	Demographic: Not reported. Socio-economic: Not reported. Psycho-emotional: Anxiety. Physical: Physical pain.	The authors emphasise the need to identify and provide appropriate care for anxiety and severe physical pain, as these negatively impact the patient and lead to threatening stress levels that must be managed, given that stress increases suicide risk. In this regard, promoting the individual’s spiritual well-being serves a protective role in severe stress situations.
Lucas et al. [52]	Mixed Study. Methods: Qualitative data were collected through structured and semi-structured interviews with an open approach. Quantitative data were gathered using standardised scales (BSI and C-SSRS). Content analysis was performed on qualitative notes, and descriptive analysis was applied to quantitative data.	Period: Not reported. Sample: 186 mother caregivers and 134 adolescent and young adult survivors (mother caregivers—women: 100%; mean age: 49.4 years; survivors—women: 43.55%; mean age: 18.18 years). Country: United States.	Demographic: Adolescents and young adults. Single status. Low educational level. Socio-economic: Low family income, recent unemployment. Psycho-emotional: Psychological distress. Physical: Not reported.	Conduct comprehensive assessments of survivors based on suicide risk. Involve family members or caregivers, providing support and access to mental health professionals when needed. Train staff in assessment tools such as BSI and C-SSRS. Implement safety protocols for vulnerable populations experiencing psycho-social distress.

SF-36: The Short Form-36 Health Survey; B-IPQ: Brief Illness Perception Questionnaire; HADS: Hospital Anxiety and Depression Scale; SRI-25: Suicide Resilience Inventory-25; SSQ: Single Suicide Question; PHQ-9: Patient Health Questionnaire-9; GAD-7: Generalised Anxiety Disorder-7; DT: Distress Thermometer; BHS: Beck Hopelessness Scale; CES-D: Center for Epidemiological Studies Depression Scale; BSS: Beck Scale for Suicidal Ideation; WHOQOL-BREF: WHO Questionnaire on Quality of Life; ECOG: Eastern Cooperative Oncology Group Performance Scale; BSI: Brief Symptom Inventory; C-SSRS: Columbia-Suicide Severity Rating Scale.

## 4. Discussion

### 4.1. Characteristics of the Studies and the Sample

From the review of the selected studies, several characteristics can be deduced. Firstly, most studies examined suicide risk in cancer patients using a quantitative approach. However, a considerable proportion of studies incorporated qualitative or mixed methods [23,43,45,46,47,48,49,50,51,52]. Other authors also highlight the relevance of qualitative approaches [53,54], as they allow a deeper exploration of the experience and capture the complexity of the phenomenon, providing an opportunity to understand meanings, emotions, perceptions, and experiences [55].

Secondly, regarding the sex of the sample, most studies included predominantly or exclusively women [23,32,33,36,37,38,39,40,42,43,45,48,50,52]. This is consistent with others research [56,57]. Nonetheless, various studies have included larger samples of men, specifying the elevated suicide risk within this population [58,59].

Thirdly, concerning the mean age of the sample, in most studies where this was described, participants were over 40 years old [31,33,35,36,37,38,39,40,48,49,52]. Several studies indicate that suicide risk increases when cancer is diagnosed after the age of 40 [60,61]. However, some studies in the review also included younger populations under 40 years [23,43,50], aligning with other authors who have reported increased suicide risk in this group [62].

### 4.2. Demographic, Socio-Economic, Psycho-Emotional, and Physical-Factors Associated with Suicide Risk in Cancer Patients

The studies included in this review identified various factors associated with suicide risk in cancer patients. Regarding demographic factors, several studies highlighted male sex, white race, older age, rural residence, and low educational attainment as relevant risks [23,31,34,35,41,50,52]. Other studies also identified adolescents and young adults as particularly vulnerable groups [37,52]. Marital status was also noted, with increased risk observed among single, divorced, and widowed individuals [41,52]. In this regard, some authors reported a significant association between being a single male cancer patient and a heightened risk of suicide [63,64].

Socio-economic risk factors were widely reported. These included lack of social support and interaction, social stigma around mental health, deterioration of family relationships, poor access to mental health services, and economic instability—all contributing to increased suicide risk among cancer patients [31,32,34,35,36,38,41,43,44,45,47,48,49,51]. Various authors highlighted the protective role of social support in enhancing resilience [65]. Others noted that higher education combined with low income may also increase suicide risk, stressing the importance of tailoring interventions to individual patient profiles [62].

Almost all studies referred to psycho-emotional factors, indicating that cancer patients often experience high emotional distress, which can lead to anxiety, depression, fear, and feelings of hopelessness. These can diminish self-efficacy and coping ability, thereby increasing vulnerability to suicidal thoughts or behaviours—especially in those with a prior history of mental illness [23,31,32,33,35,36,37,38,39,40,41,42,43,44,45,46,47,48,49,51,52]. These findings are consistent with the existing literature, which stresses the role of psychological state in the clinical progression of cancer, with anxiety and depression being key risk factors for suicidal ideation [62,66].

Most studies also addressed physical factors, describing common cancer-related symptoms such as fatigue, weakness, and especially pain as major sources of distress and suffering. These symptoms can contribute significantly to suicidal risk [23,31,32,33,34,35,36,40,42,44,45,46,47,48,49,50,51]. Some authors emphasised the importance of strengthening physical care strategies from the point of diagnosis, highlighting the strong correlation between unmanaged symptoms (particularly pain) and lower quality of life, which may increase the risk of suicide in this population [67,68].

### 4.3. Nursing Care to Reduce the Suicide Risk in Oncology Patients

Multiple studies emphasise the crucial role of oncology nurses in assessing suicide risk, delivering person-centred interventions, and coordinating care within multidisciplinary teams [35,46,47,48,50,52]. With regard to nursing care strategies, the following aspects should be considered:

(1) Systematic suicide risk assessment: Validated tools such as the C-SSRS and the Plutchik Risk Scale are essential for early detection and continuous monitoring of suicide risk [31,33,34,35,36,37,41,42,43,44,45,46,47,48,49,50,52]. Clinical practice guidelines recommend applying these tools regularly—particularly after critical changes in diagnosis or psycho-social circumstances [35,48]. In nursing settings, this involves embedding screening into electronic health records and integrating assessments into routine consultations, as suggested by Gascón et al. [25] and Janes [69].
(2) Professional training and communication skills development: Continuous professional development is crucial to improve nurses’ ability to recognise suicidal ideation, express empathy, and build trust with patients [35,36,39,40,43,44,45,46]. This education should be grounded in an understanding of associated risk factors and clear knowledge of appropriate response protocols [70,71]. Simulation-based training and structured communication models—such as KoMPASS—have demonstrated success in improving detection and intervention rates in clinical settings. From a practice standpoint, this means institutional investment in annual suicide prevention workshops, role-play exercises, and the creation of ethical guidelines for addressing existential distress [39]. Richard et al. [71] highlight the importance of simulation as a pedagogical tool in enhancing confidence and competence in suicide risk management.
(3) Therapeutic and supportive nursing interventions: Interventions such as progressive muscle relaxation, therapeutic walking, aromatherapy, and problem-solving therapy reduce distress and improve patient well-being. Nurses can incorporate these into individualised care plans, especially in palliative and chemotherapy settings where physical symptoms and emotional fatigue often co-occur [40,43,45]. Liu et al. [72] confirm that aromatherapy via inhalation significantly decreases anxiety and depression levels in cancer patients, while Bosman et al. [73] show the utility of art-based interventions in supportive care.
(4) Integration into routine clinical practice and interdisciplinary coordination: Risk detection protocols are most effective when integrated into daily nursing documentation and shift handovers. Collaboration with mental health professionals and social workers is vital to ensure rapid intervention in high-risk cases. In clinical terms, this translates to clear referral pathways, shared decision-making protocols, and multidisciplinary case reviews [35,41,46]. Studies outside the review support these findings, emphasising the importance of multidimensional care, particularly during times when patients may have more complex or shifting needs [73,74]. Kobayakawa et al. [75] advocate for the inclusion of mental health specialists early in the care continuum to address emotional crises and coordinate support for both patients and families.
(5) Family and caregiver support: Suicide prevention must extend beyond the patient to include family members and caregivers, who may also be experiencing psychological distress. Nurses should be trained to screen caregivers for burden and emotional fatigue, facilitate emotional support sessions, and provide referrals to psychological services when needed [32,38,47,48,49,50,51,52]. Lucas et al. [52] demonstrate the effectiveness of caregiver support in reducing distress and improving long-term psycho-social outcomes. In practice, this can involve family-centred care plans, psycho-educational materials, and structured communication strategies to reinforce the family’s protective role.

### 4.4. Key Implications for Clinical Practice, Health Policy, and Future Research

(1) Relevance for clinical nursing practice: 1. Incorporate validated tools such as the Columbia-Suicide Severity Rating Scale (C-SSRS) and the Plutchik Suicide Risk Scale into routine nursing assessments for systematic suicide risk screening [31,35,44,48,50,52]. 2. Promote non-pharmacological nursing interventions—such as progressive muscle relaxation, therapeutic walking, and aromatherapy—to alleviate physical and emotional distress [40,43,45]. 3. Strengthen empathic communication and patient-centred care through active listening, identification of distress signals, and therapeutic nurse–patient relationships [23,39,44,47].
(2) Implications for institutional and health policy: 1. Develop and implement ongoing professional training programmes on suicide prevention, including communication skills, ethical protocols, and clinical simulation methods (e.g., the KoMPASS programme) [35,39,45]. 2. Integrate suicide risk screening into electronic nursing documentation and standardise referral pathways through interdisciplinary coordination with mental health professionals and social workers [35,41,45,46].
(3) Priorities for future research: 1. Conduct controlled studies to evaluate the effectiveness of specific nursing interventions—such as relaxation techniques, family support strategies, and structured psycho-social care—in suicide prevention [32,43,45,52]. 2. Undertake longitudinal research to identify high-risk periods across the cancer care continuum, especially among adolescents, young adults, and caregivers [37,52]. Explore the cultural adaptability and global applicability of these nursing approaches by including under-represented populations and diverse healthcare systems [19,34].

### 4.5. Limitations

This review presents several limitations: Firstly, the study design itself, as one inherent limitation of literature reviews is the inability to retrieve all existing information on a given topic [30]. Nevertheless, a rigorous and thorough process was followed to obtain the best available scientific evidence. Secondly, the lack of use of tools to assess the quality of the selected studies may also compromise and limit the validity of the results [30]. While quality appraisal would have provided valuable information to interpret the strength and potential biases of the evidence, the heterogeneity of study designs and methodologies made it challenging to apply a uniform and appropriate appraisal tool. Thirdly, the inclusion of quantitative, qualitative, and mixed-methods studies may introduce a degree of heterogeneity in the results; however, it enables a more comprehensive understanding of the phenomenon. The triangulation, complementarity, and expansion among the different approaches strengthen the thematic robustness while allowing for more solid inferences by contextualising both quantitative and qualitative findings [76,77]. Another relevant limitation is the potential geographical bias arising from the origin of the studies, which are predominantly based in Asia and North America. This may affect the applicability of the findings in countries with different cultural, ethical, and healthcare frameworks. Encouraging research in other regions would be desirable in order to obtain a more global and diverse perspective on the nursing approach to suicide risk among patients with cancer [19,78].

## 5. Conclusions

Suicide risk in cancer patients is influenced by multiple interrelated factors, including demographic, socio-economic, psycho-emotional, and physical variables. This review identified male sex, advanced age, lack of social support, chronic pain, fatigue, and a history of psychiatric disorders as key risk factors.

In terms of prevention, the following nursing intervention strategies have been identified: (1) regular suicide risk assessment using validated tools (e.g., C-SSRS, Plutchik); (2) continuous training for nurses in communication and suicide risk management; (3) implementation of therapeutic techniques such as muscle relaxation and walking therapy; (4) institutional protocols to integrate risk screening into nursing documentation and workflows; and (5) psychological support and suicide risk monitoring for family members and caregivers.

These findings reinforce the essential role of oncology nurses in suicide prevention and provide a practical framework for designing and implementing nursing interventions aimed at reducing suicide risk throughout the cancer care continuum.

## Data Availability

Not applicable.

## Public Involvement Statement

No public involvement in any aspect of this research.

## Guidelines and Standards Statement

This manuscript was drafted following the recommendation of Urrútia & Bonfill [28].
